# The affect of goserelin on the QoL of women having chemotherapy for EBC: Results from the OPTION trial

**DOI:** 10.1016/j.breast.2020.05.009

**Published:** 2020-05-29

**Authors:** Leonard R, Yellowlees A, Mansi J, Fallowfield L, Jenkins V

**Affiliations:** a Department of Surgery and Cancer, Imperial College, London, United Kingdom; b Quantics Consulting Ltd, Edinburgh, United Kingdom; c Department of Oncology, Guy’s and St Thomas’ NHS Foundation Hospital, London, United Kingdom; d Sussex Health Outcomes Research & Education in Cancer (SHORE -C) University of Sussex, United Kingdom

**Keywords:** OPTION trial, Pre-menopausal, Breast cancer, Goserelin, Chemotherapy, Quality of life

## Abstract

**Background:**

The OPTION trial results showed that premenopausal women with early stage breast cancer (EBC) receiving chemotherapy benefited from ovarian function protection with goserelin. The impact of treatments on patient reported Quality of Life (QoL) were also examined.

**Patients and methods:**

227 pre-menopausal women with EBC, were randomly assigned to chemotherapy±goserelin (C±G); 132 (58%) were ER-ve. Patients were stratified by age (≤40 years and >40 years). QoL was assessed with the Functional Assessment of Cancer Therapy – Breast, and Endocrine Symptom checklist at baseline (pre-treatment), 3, 6, 12, 18 and 24 months, then annually to 5 years. Treatment Outcome Index (TOI) score was the primary outcome.

**Results:**

213 patients were available for QoL analysis. There was a significant decrease in TOI scores for both treatment groups at 3 and 6 months that returned to pre-treatment levels at 12 months, then continued to increase reflecting improved QoL. By 3 months there was a significant difference from baseline in both groups for menopausal symptoms, and between groups in the proportion experiencing hot flushes at any time. The C + G group experienced higher levels of vasomotor symptoms generally during the treatment phase; by 24 months, the short-term negative effect of goserelin was reversed, with hot flushes twice as frequent in the chemotherapy only group (40.9% vs 21.3%).

**Conclusions:**

These results show that young women diagnosed with breast cancer experienced only a short-term decrease in QoL from the addition of goserelin, in order to preserve ovarian function during chemotherapy treatment.

## Highlights

• Ovarian damage from breast cancer chemotherapy is a concern for the younger patient.
• OPTION showed goserelin reduced the rate of premature ovarian insufficiency in the ≤40yrs.
• Patient reported QoL is an important consideration in evaluating these treatments.
• OPTION QoL revealed greater vasomotor symptoms with goserelin at 3 and 6 mths.
• Overall goserelin treatment resulted in only a short term decrease in QoL.

## 1 Introduction

The improved survival of women with early breast cancer (EBC) has led to an increased interest in the long-term consequences of treatment. Chemotherapy treatment-related side effects concern women regardless of age, e.g. fatigue, hair loss, but it is the impact of ovarian damage from chemotherapy that is a specific issue for the premenopausal patient [1]. The harm from treatments range from follicular damage with preserved menses to temporary amenorrhoea, irregular menses, and early menopause (premature ovarian insufficiency, POI) resulting in a loss of fertility. Recall of menses may be an unreliable indicator of POI unless based on a daily diary, and while amenorrhoea is clear, infrequent, or irregular menses may indicate incipient POI [2].

These young women with breast cancer face unique challenges. Efforts to protect fertility include freezing embryos (IVF), eggs or ovarian tissue before starting treatment, and the use ovarian suppression [3]. In these circumstances, some women will have a plan, while others may find it harder to decide and prefer to start chemotherapy and wait to see if fertility returns when treatment is over [4]. A European prospective study (Helping Ourselves, Helping Others) revealed that 64% of young breast cancer patients were very concerned about becoming infertile after chemotherapy [1]. In others, 68% reported sexual dysfunction, and 58% concerns over fertility two years post diagnosis, and sexual problems [5,6].

One strategy to reduce the risk of treatment induced premature menopause includes suppressing the ovaries temporarily with a gonadotropin-releasing hormone agonist (GnRHa), but data from randomised controlled trials (RCTs) are mixed. Many of the studies included a limited number of patients and reported results of their primary endpoint (i.e. chemotherapy-induced POI) rather than successful post treatment pregnancies. A review of 873 patients from five trials noted that overall POI rate was 14.1% in the GnRHa group and 30.9% in the control group (adjusted odds ratio, 0.38; 95% CI, 0.26 to 0.57; P < 0.001) [7]. A total of 37 (10.3%) patients had at least one post-treatment pregnancy in the GnRHa group and 20 (5.5%) in the control group. Importantly no significant differences in disease-free survival and overall survival were observed between the groups. These data provided evidence for the safety of temporary ovarian suppression with GnRHa during chemotherapy as an available option to reduce the likelihood of chemotherapy-induced POI, and potentially improve future fertility in premenopausal patients with EBC.

The OPTION trial was set up to establish specifically if the use of goserelin in women who require chemotherapy for operable hormone-*insensitive* breast cancer or for whom ovarian suppression is not considered a necessary part of treatment, may reduce the risk of POI [8]. The primary outcome was the prevalence of amenorrhoea at 12–24 months, secondarily combined with elevated follicle-stimulation hormone (FSH) concentration giving the prevalence of POI. The original protocol restricted the entry of patients to those with ER negative tumours only, but patients with ER positive tumours for whom the investigator did not deem ovarian suppression necessary as part of the treatment, were allowed entry to the trial after a protocol amendment.

A total of 227 patients were randomised, and the primary analysis was conducted on 202 patients. Goserelin reduced the prevalence of amenorrhoea between 12 and 24 months to 22% versus 38% in the control group (P = 0.015) and the prevalence of POI to 18.5% versus 34.8% in the control group (P = 0.048). Follicle stimulating hormone concentrations were also lower in all women treated with goserelin at both 12 and 24 months (P = 0.027, P = 0.001, respectively). Assessment of the ovarian reserve using anti-Müllerian hormone showed a marked fall in both groups during treatment to median values of 5% of pre-treatment levels in the control group, and 7% in the goserelin group, which were not significantly different between groups. Results showed both outcomes were significantly reduced in patients receiving goserelin, but only in younger patients, aged up to 40 years at randomisation [2]. Despite these benefits, POI has potential adverse consequences, including decreased quality of life [9], sexual dysfunction [5,6], and menopausal symptom distress [10] and in the longer term women are at increased risk of developing osteoporosis, and cardiovascular disease [11,12].

The impact from POI can be so great that both ESMO [10,13], ASCO [14] issued international guidelines, advising that risk and approaches to reduce the associated side effects are discussed as early as possible with all young women considering chemotherapy treatments. Given the potential side effects associated with POI, it is surprising that patient reported outcomes (PROs) were rarely incorporated into the earlier trials. OPTION was one of the few that collected Quality of Life (QoL) PRO data on the immediate and late effects of chemotherapy±goserelin treatments. These data are presented here to help inform patients and health care professionals when discussing fertility preservation options.

## 2 Methods

### 2.1 Participants

Premenopausal patients with histologically confirmed EBC who were to receive adjuvant or neo-adjuvant chemotherapy were eligible for OPTION. Metastatic disease was an exclusion criterion. Patients who had had prior chemo or endocrine therapy were ineligible. Participants were randomised to receive a 3.6-mg goserelin implant or nothing, starting at least 1 week but preferably 2 weeks before chemotherapy. Goserelin treatment continued 3–4 weekly until the end of the chemotherapy, which had to start within 8 weeks of definitive surgery. Radiotherapy was as per standard protocol for each centre. Participants kept a menstruation diary for 24 months from the start of chemotherapy. The detailed description of the trial design was published in 2017 [2].

### 2.2 Quality of life measures

Patients recruited to the OPTION trial completed the Functional Assessment of Cancer Therapy FACT-B [15], a standardised breast cancer quality of life measure, together with the Endocrine Subscale (FACT-ES) [16].

The FACT–B is a 36-item questionnaire that measures both general QoL associated with cancer (27 questions, referred to as the FACT-General [FACT–G]), and additional concerns more specific to women with breast cancer (nine items, referred to as the breast cancer subscale). The ES was designed for use with the FACT–B and comprises 19 items.

The FACT ES has 4 subscales, Physical Well Being (PWB) 7 items, Social Well Being (SWB) 7 items, Emotional Well Being (EWB) 6 items, Functional Well Being (FWB) 7 items and a 19-item ES. Participants indicated, using a five-point Likert scale ranging from 0 (not at all), 1 (a little bit), 2 (somewhat), 3 (quite a bit), to 4 (very much), to what degree each item has applied over the last 7 days. High scores equate with a good QoL and lower scores equate with a poorer QoL. The tools sit within the Functional Assessment of Chronic Illness Therapy (FACIT) measurement system [17].

Baseline questionnaires were completed by patients without assistance prior to randomisation with follow-up assessments at 3, 6, 12, 18, 24 months after therapy started; and then yearly thereafter until 5 years (i.e. 36, 48, 60 months).

### 2.3 Statistical methods

The primary endpoint was the Treatment Outcome Index (TOI) score: this comprised the 23 items of the Physical and Functional well-being and Breast Cancer Subscale. Secondary endpoints were-

1) FACT-ES score: total of all 36 + 19 = 55 items
2) ES score: total of 19 Endocrine symptom scores
3) Individual endocrine symptom scores

For TOI, ES and FACT-ES scores, change from baseline to each time point was calculated for all patients with valid baseline and follow-up questionnaires; no imputation was made for missing data.

Mixed model repeated measures analyses were performed where the dependent variable was the change from baseline, the fixed factors were time point, treatment and the interaction of time with treatment, and the baseline value of the endpoint was included as a covariate in the model. A random effect was included for patients. The model assumed a first order autoregressive correlation structure. The estimate of the treatment term provides an overall measure of treatment difference for the whole period of 3–60 months. Where the interaction term is significant, there is evidence that the treatment difference varies across time points. Where neither the treatment term nor the interaction term is significant, there is no evidence of a treatment effect at any time point. At each time point, 95% confidence intervals for the change from baseline, based on the repeated measures model, were calculated for each treatment group.

The 19 endocrine symptoms form four conceptually meaningful clinical categories: A) Vasomotor symptoms, B) Neuropsychological symptoms, C) Gastro-intestinal (GI) symptoms and D) Gynaecologic symptoms. ‘Joint Pains’ was presented separately. For each individual symptom score at each time point, a binary variable was created indicating ‘Clinically significant effect’, defined as a response of ‘Very much’ or ‘Quite a bit’. These data were summarised as numbers and percentages for each symptom and each time point, plus an exact 95% confidence interval for the percentage (calculated using the Clopper Pearson method).

In addition, a binary variable for each symptom score was created indicating whether or not a patient reported a clinically significant effect at any time; the odds ratios (OR) for this binary variable was calculated for each symptom plus an exact 95% confidence interval for the percentage as above. ORs less than 1 imply a better QoL for the treatment including goserelin: i.e. the odds of experiencing the symptom were lower in the group treated with goserelin. Forest plots showing this OR, with a 95% confidence interval (CI) were created.

The analysis was conducted using R [18].

## 3 Results

Two hundred and twenty-seven patients were randomised between August 26, 2004 and December 23, 2009. The age distribution, ER status, definitive surgery, planned chemotherapy cycles and hormone levels for the 227 patients randomised are described in Table 1, and did not differ between the two groups, of these 213 completed at least one QoL questionnaire. Table 2 shows the number of QoL questionnaires completed at each follow up time point. Completion rates and attrition were similar between groups.

**Table 1:** Demographics and baseline characteristics (all randomised).

			Chemotherapy plus goserelin	Chemotherapy
		N	106	121
Age		n	106	121
		Mean (SD)	37.7 (5.57)	38.2 (5.40)
		Min, Max	25, 50	24, 51
		≤40	73 (68.9%)	77 (63.6%)
		>40	33 (31.1%)	44 (36.4%)
ER status	% negative	n	106	121
		n (%)	63 (59.4%)	69 (57.0%)
Surgery pre randomisation	Conservation	n	106	121
		n (%)	55 (51.9%)	59 (48.8%)
	Mastectomy	n	106	121
		n (%)	29 (27.4%)	28 (23.1%)
Planned chemo cycles		n	106	119
		Mean (SD)	6.8 (0.99)	6.7 (1.00)
		Min, Max	6, 8	4, 8
Oestradiol pmol/l^1^	Before chemo	n	91	105
		Mean (SD)	662.2 (1628.52)	486.4 (1130.44)
		Min, Max	20, 13774	1.49, 11190
	After chemo	n	51	50
		Mean (SD)	666.4 (1039.07)	593.9 (1045.70)
		Min, Max	29, 4371	23, 5476
FSH U/l^a^	Before chemo	n	99	108
		Mean (SD)	6.22 (5.579)	9.09 (9.846)
		Min, Max	1.1, 35.2	0.5, 67.8
	After chemo	n	64	68
		Mean (SD)	26.03 (21.032)	42.66 (31.652)
		Min, Max	2.2, 84.9	2.9, 120.7
LH U/l^1^	Before chemo	n	96	106
		Mean (SD)	5.54 (4.980)	11.06 (32.124)
		Min, Max	0.4, 32.7	0.1, 329
	After chemo	n	64	69
		Mean (SD)	17.64 (12.001)	27.77 (29.055)
		Min, Max	0.9, 46.1	1, 222

a Bounded values (such as “<50” or “>50”) have been treated as missing. The occurrence of such values is 6 (Oestradiol, before chemo), 33 (Oestradiol after chemo), 0 (FSH, before chemo), 4 (FSH, after chemo), 5 (LH, before chemo), and 1 (FH after chemo).

**Table 2:** Quality of Life Questionnaires completed at each time point.

		Randomised	Responses at each time point (months)								
			0	3	6	12	18	24	36	48	60
All	N	227	203	193	186	178	169	163	114	83	40
	%^a^		89%	85%	82%	78%	74%	72%	50%	37%	18%
Chemotherapy	N	106	96	91	84	83	79	75	57	41	22
plus goserelin	%		91%	86%	79%	78%	75%	71%	54%	39%	21%
Chemotherapy	N	121	107	102	102	95	90	88	57	42	18
	%		88%	84%	84%	79%	74%	73%	47%	35%	15%

a Percentage of randomised.

### 3.1 TOI results

Table A1 (Appendix A) provides summary statistics for the TOI at each time point. Table A2 provides the observed mean change from baseline and the least squares means and 95% confidence intervals for the change from baseline in TOI at each time point calculated from the repeated measures model.

**Fig. 1:**
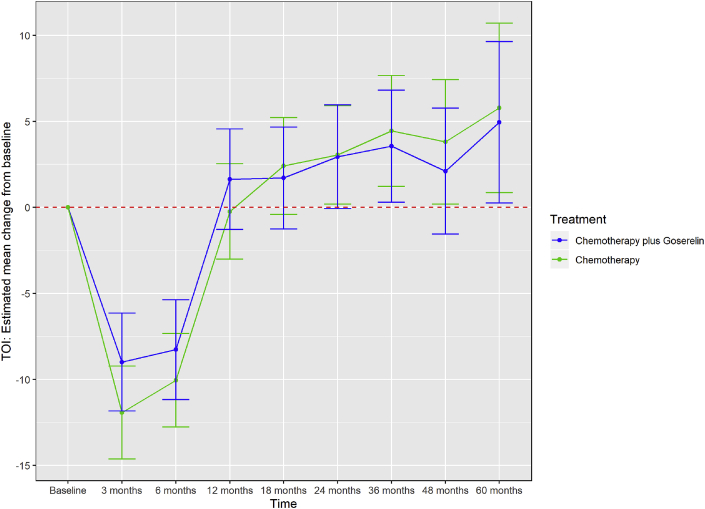
Estimated Mean TOI change from baseline with 95% CIs (from repeated measures model).

Fig. 1 illustrates the change from baseline for TOI for each treatment group. In the mixed model analysis, time point was a significant factor in the model (P < 0.001), but neither treatment group (P = 0.56) nor the interaction of treatment with time (P = 0.60) was significant.

There was no evidence of any difference between the treatment groups. For both treatment groups, there were significant decreases in the TOI at both 3 months and 6 months, corresponding to the period of chemotherapy treatment. TOI returned to pre-treatment levels at 12 months, then continued to increase, with higher estimated scores than pre-treatment from 12 months onwards (C + G) and respectively from 18 months onwards (C only). For patients treated with C only the increase in estimated scores was significant from 24 months onwards, while for patients treated with C + G the increase was significant at 36 months and at 60 months. (This should not be interpreted as a difference between the treatment arms; data are limited in the later part of the study.)

### 3.2 ES results

**Fig. 2:**
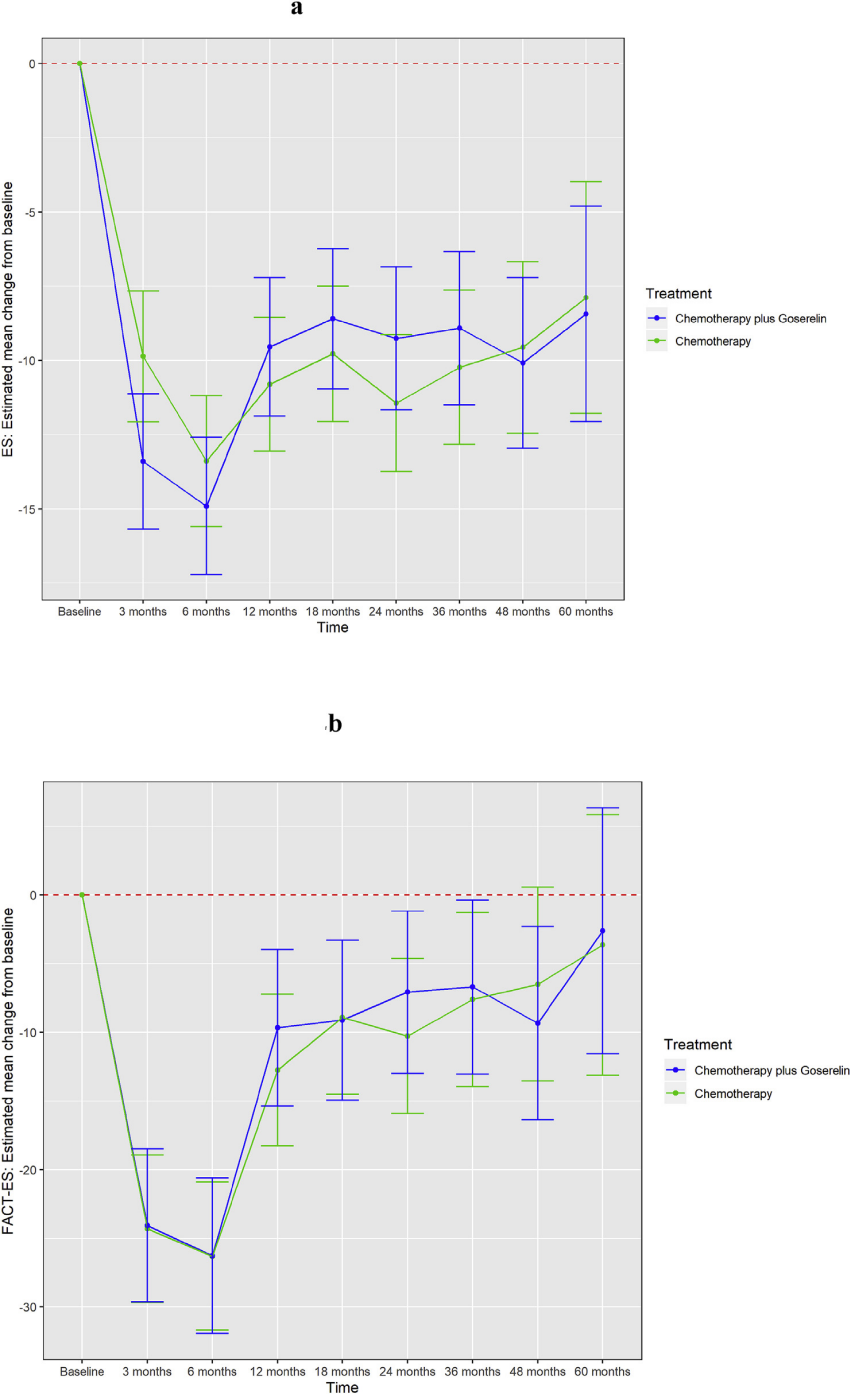
a: Estimated Mean Endocrine Symptom (ES) change from baseline with 95% CIs (from repeated measures model) b: Estimated Mean FACT-ES change from baseline with 95% CIs (from repeated measures model).

Fig. 2a and Tables B1 and B2 (Appendix A) show the summary statistics for the ES scores and the least squares means and 95% confidence intervals for the changes from baseline at each time point. In the mixed model analysis, time point was a significant factor in the model (P < 0.001). Treatment group was not significant overall (P = 0.74) but the interaction of treatment with time was marginally significant (P = 0.02) suggesting that the treatment effect may be time dependent. The figure suggests an earlier worsening of symptoms in the goserelin group. The pattern was similar to that seen for the TOI, with a reduction from baseline at 3 and 6 months followed by an upward trend; however at 60 months the values had not returned to pre-treatment levels although the data from this time point are limited.

### 3.3 FACT-ES total score results

The FACT-ES total scores were again similar between the treatment groups for each of the time points (Fig. 2b; Tables C1, C2, Appendix A). In the mixed model analysis, time point was a significant factor in the model (P < 0.001), but neither treatment group (P = 0.81) nor the interaction of treatment with time (P = 0.74) was significant. By 60 months the values had returned to pre-treatment levels.

### 3.4 FACT-ES symptoms

**Fig. 3:**
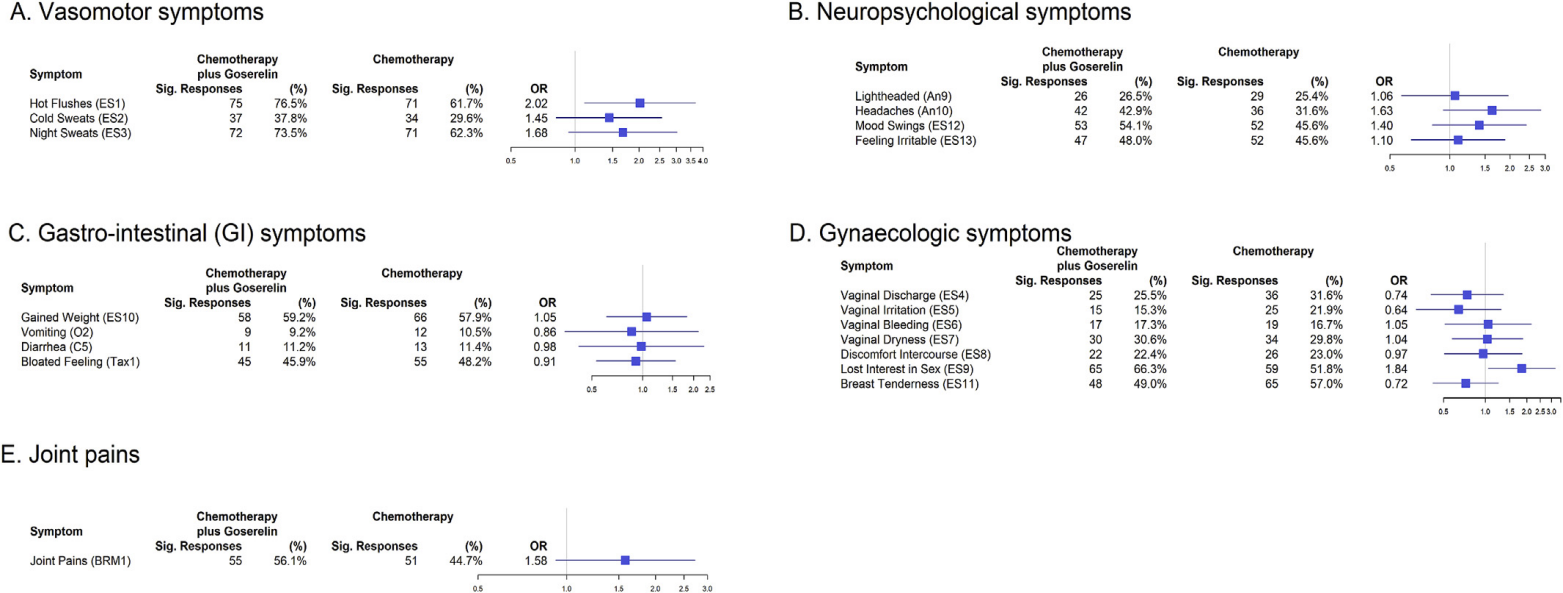
Odds ratios (ORs) with 95% CIs and proportions of patients reporting clinically significant symptoms at any time.

Fig. 3 shows the forest plots for the odds ratios comparing the treatment groups for the proportions of patients reporting each symptom at a clinically significant level (i.e. ‘very much’ or ‘quite a bit’) at any time. ORs less than 1 imply a better QoL for the treatment including goserelin: i.e. the odds of experiencing the symptom being lower in the group treated with goserelin. Considerable proportions of patients in each treatment group reported that at some point, hot flushes, cold and night sweats or joint pains had affected them quite a bit or very much. A larger proportion of patients in the C + G group experienced clinically significant vasomotor symptoms compared to those receiving C only. There was a statistically significant difference between groups in proportions experiencing significant hot flushes (76.5% C + G group, 61.7% C group: odds ratio 2.02 (95% confidence interval (1.11, 3.68)).

Fig. 3 also shows that there were no statistically significant differences between groups in proportions of patients reporting each neuropsychological symptom or in reporting each GI symptom at any time. Proportions experiencing clinically significant symptoms were marginally higher in the C only group except for weight gain, where a slightly higher proportion of patients in the C + G group experienced clinically significant symptoms. There was a statistically significant difference between groups for loss of interest in sex with 66.3% in the C + G group and 51.8% in the C only group reporting this at any time point (odds ratio 1.84 (95% confidence interval (1.05, 3.21)).

**Fig. 4:**
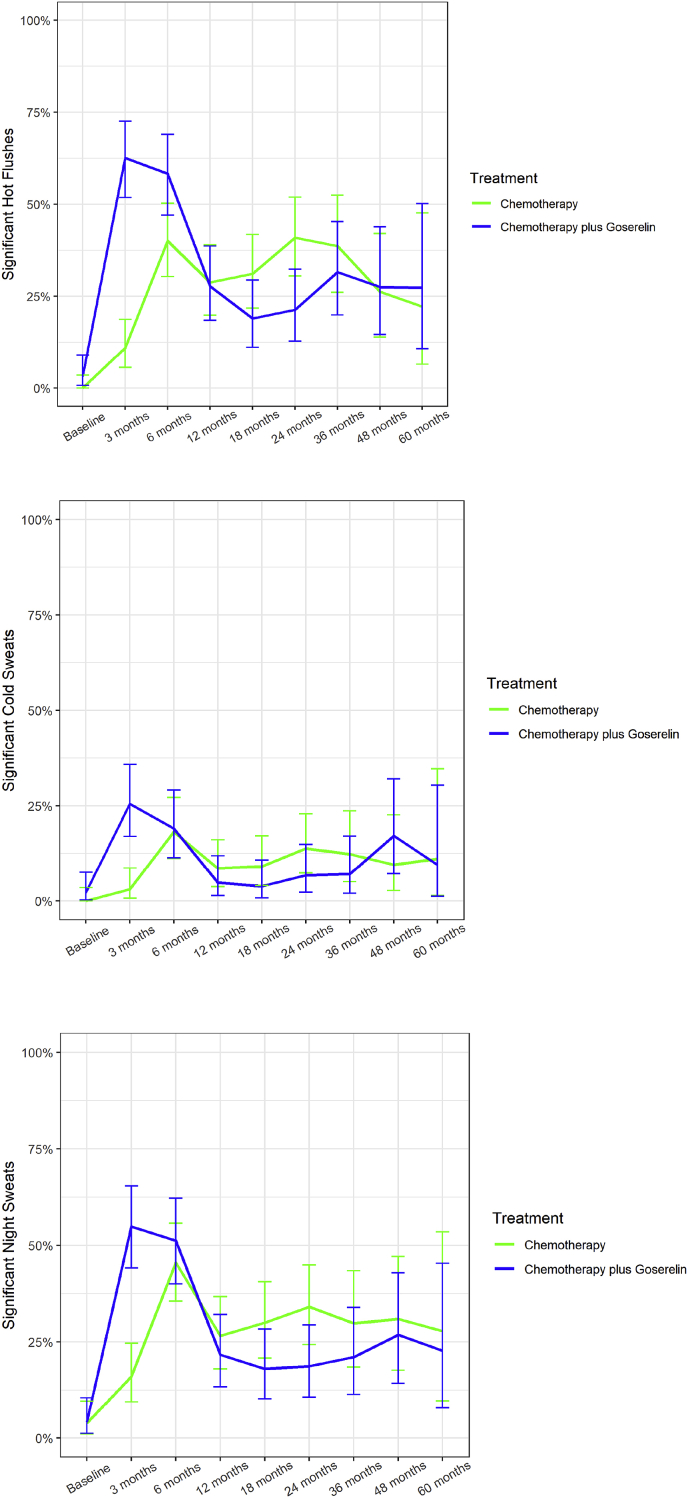
Proportions reporting clinically significant vasomotor symptoms (95% CIs: Clopper Pearson).

Fig. 4 shows the proportions of patients reporting clinically significant vasomotor symptoms at each time point (hot flushes; cold sweats and night sweats). At 24 months, when the main effect of goserelin was observed in respect of menses returning, the short-term (3–6 months) negative effect of goserelin was reversed with hot flushes being twice as frequent in the C only group (40.9% (95% confidence interval (30.5%, 51.9%) vs 21.3% (95% confidence interval (12.7%, 32.3%)).

## 4 Discussion

The OPTION sub study examined QoL in women receiving chemotherapy with or without a reversible suppressor of ovarian function (goserelin). During treatment there was an increase in some endocrine symptoms in both groups and an associated deterioration in overall QoL by three months, which gradually started to improve after six months but did not return to pre-treatment levels for most women.

There was evidence of a difference between the groups during the treatment phase, with a larger incidence of vasomotor symptoms in the goserelin group. The drug had an immediate impact on vasomotor endocrine symptoms as compared to the chemotherapy-only group where these symptoms are likelier to evolve more slowly, and possibly incompletely, as not every patient who has chemotherapy experiences a permanent menopause. However, by 24 months the pattern had reversed and higher proportions of women who had received chemotherapy-only were reporting bothersome endocrine symptoms. The finding was consistent with the clinical trial analysis where 38.3% of the chemotherapy-only group became menopausal due to the chemotherapy; proportions differed according to age group (those over 40 years 54.2%; those 40 years and under 25.4%) [2].

It is difficult to compare the OPTION QoL results directly with other breast cancer ovarian suppression trials as some did not include patient reported outcomes (PROs), for example POEMS S0230 (Prevention of Early Menopause Study) [19]. Others e.g. SOFT (Suppression of Ovarian Function) and its sister trial TEXT (Triptorelin with either EXemestane or Tamoxifen) involved pre-menopausal women with hormone sensitive breast cancer not all of whom received chemotherapy. These trials used the International Breast Cancer Study Group (IBCSG) QoL core form to collect PRO data at baseline, every 6 months for 2 years and then every year in years 3–6. PRO analysis involved changes from baseline on the global and symptom indicators, across time. Results showed that ovarian function suppression (OFS) with tamoxifen or exemestane impacted on QoL with distinct effects of the two treatments on endocrine symptoms [20]. Subsequently analysis of a subset of patients showed that treatment induced symptoms predicted sexual problems during the first 2 years [6].

Another study, MENOCOR, examines the impact of chemotherapy induced menopause (CIM) on QoL [9]. Preliminary results from 58 women (age, 18–46 years) show that overall QoL, perhaps not surprisingly, differed significantly for both CIM (n = 41) and non-CIM (n = 17) groups at six months post chemotherapy. Menopausal symptoms however were greater in the CIM group who were significantly older. In OPTION the rate of menopause overall for women in the goserelin group was 22.1%; 42.9% in those >40 years and 10% ≤ 40years. In the QoL analysis we did not attempt to look at the age sub-groups separately but it would be reasonable to assume that for the younger women, short term decline in QoL from goserelin is counterbalanced by a longer term gain in association with preserved ovarian function.

Limitations of our study include the modest size of the ≤40years age group, where the largest effects of ovarian protection were always likeliest to be strongest but results still give an insight into the affect ovarian suppression has on QoL. In addition, subgroup analysis for the differing effect of endocrine treatment on QoL (tamoxifen for premenopausal, aromatase inhibitors for post-menopausal) would have been futile as over the 5 years, more women became postmenopausal and others possibly switched treatments. There is always a challenge collecting longitudinal QoL clinical trial data, with missing data and a drop in return rates over time. This was true for the OPTION QoL sub study, and we have no information on the reasons why, but missing data were balanced between groups, allowing us confidence in our comparison results.

Finally, a 1980’s study on Hodgkin’s lymphoma in young women treated by MVPP (mustine, vinblastine, procarbazine, and prednisolone) chemotherapy may be instructive. Most of these significantly younger patients recovered menstruation in their 20’s only to develop menopause in their mid 30’s, many years prematurely [21]. A similar pattern of delayed ovarian failure in breast cancer patients would justify protracted follow-up to manage the bone health and other sequelae of premature menopause.

Our data suggest that women experience a short-term decrease in QoL from the addition of goserelin to chemotherapy in order to preserve ovarian function. These findings should give hope to the many young women with breast cancer who worry about the negative impact of treatments on fertility. Results should also help Health Care Professionals to adequately inform young women at the time of diagnosis about the risk of infertility and the different available methods for fertility preservation, including the use of goserelin.

## Funded

10.13039/501100000289Cancer Research UK who had no role in the collection, analysis and interpretation of data; in the writing of the report; and in the decision to submit the article for publication. The Quality of Life data used in this analysis are stored with Public Health Scotland and an anonymised set are available on permission by written request to the custodian – Public Health Scotland.

## Declaration of competing interest

The authors have no financial relationships relevant to the submitted work.

Details of Ethics Approval: The study was approved by the North West research ethics (ref MREC/03/6/90).

## Figures and Tables

**Table A1:** Trial Outcome Index observed score

	Chemotherapy plus Goserelin						Chemotherapy					
	N	Mean	SE^3^	Median	Min	Max	N	Mean	SE	Median	Min	Max
Baseline	95	65.01	1.22	67.00	24.00	89.88	107	65.36	1.27	66.00	25.00	89.00
3 months	91	55.58	1.67	55.00	15.00	91.00	102	53.51	1.57	53.50	22.00	86.00
6 months	84	57.10	1.83	56.50	19.00	92.00	102	55.26	1.70	55.50	15.00	90.00
12 months	83	66.42	1.63	67.00	30.00	91.00	95	65.60	1.56	69.00	28.00	90.00
18 months	79	67.94	1.60	70.00	23.00	89.00	90	68.69	1.66	72.50	17.67	90.00
24 months	75	68.66	1.83	73.00	28.00	92.00	87	68.86	1.71	72.00	19.00	91.00
36 months	57	70.78	2.39	78.00	20.00	92.00	57	70.68	1.88	74.00	27.00	90.00
48 months	41	69.48	2.67	75.00	27.75	90.00	42	70.83	2.51	76.94	29.00	91.00
60 months	22	74.56	3.23	82.00	44.25	92.00	18	75.06	4.06	80.00	30.00	90.00

3 SE – standard error of the mean.

**Table A2:** Trial Outcome Index change from baseline

	Chemotherapy plus Goserelin					Chemotherapy				
		Observed	Estimated				Observed	Estimated		
	N	Mean	Mean	95% LCL^4^	95% UCL^5^	N	Mean	Mean	95% LCL	95% UCL
3 months	90	−9.31	−8.99	−11.84	−6.14	98	−11.82	−11.93	−14.64	−9.23
6 months	83	−7.91	−8.27	−11.17	−5.37	96	−9.66	−10.05	−12.76	−7.34
12 months	82	2.11	1.64	−1.28	4.55	89	0.49	−0.24	−3.01	2.53
18 months	78	2.66	1.71	−1.26	4.67	85	3.55	2.41	−0.41	5.23
24 months	74	3.41	2.94	−0.07	5.95	82	4.35	3.05	0.20	5.91
36 months	55	4.75	3.56	0.30	6.81	53	6.16	4.44	1.22	7.67
48 months	40	3.58	2.11	−1.55	5.77	40	6.88	3.81	0.18	7.43
60 months	21	11.27	4.94	0.25	9.64	18	8.00	5.78	0.85	10.71

4 LCL = lower confidence limit.

5 UCL = upper confidence limit.

**Table B1:** Endocrine Symptoms observed score

	Chemotherapy plus Goserelin						Chemotherapy					
	N	Mean	SE	Median	Min	Max	N	Mean	SE	Median	Min	Max
Baseline	95	67.26	0.80	69.00	40.00	76.00	104	67.47	0.76	69.00	33.00	76.00
3 months	91	53.54	1.20	55.88	28.00	74.00	100	57.59	1.11	59.50	19.00	76.00
6 months	84	52.67	1.30	55.00	24.00	74.00	101	53.65	1.31	56.00	18.00	76.00
12 months	83	57.39	1.24	60.00	20.00	76.00	94	57.32	1.23	60.00	24.00	76.00
18 months	79	58.92	1.40	62.00	14.00	76.00	90	58.72	1.33	62.00	24.00	76.00
24 months	75	58.20	1.39	59.00	26.00	76.00	88	56.62	1.39	58.53	22.00	75.00
36 months	57	59.45	1.65	61.00	27.00	76.00	57	57.96	1.79	61.00	22.00	76.00
48 months	41	58.57	2.11	62.00	20.00	75.00	42	59.61	2.01	62.64	31.00	76.00
60 months	22	60.76	2.89	64.50	27.31	76.00	18	60.67	2.71	63.00	30.00	75.00

**Table B2:** Endocrine Symptoms change from baseline

	Chemotherapy plus Goserelin					Chemotherapy				
		Observed	Estimated				Observed	Estimated		
	N	Mean	Mean	95% lcl	95% ucl	N	Mean	Mean	95% lcl	95% ucl
3 months	90	−13.56	−13.39	−15.67	−11.12	94	−9.89	−9.87	−12.06	−7.67
6 months	83	−14.39	−14.90	−17.22	−12.58	92	−13.27	−13.38	−15.59	−11.18
12 months	82	−9.27	−9.54	−11.87	−7.21	85	−10.39	−10.80	−13.05	−8.55
18 months	78	−8.29	−8.60	−10.96	−6.24	82	−8.75	−9.78	−12.06	−7.50
24 months	74	−8.74	−9.25	−11.65	−6.86	80	−11.14	−11.43	−13.73	−9.13
36 months	55	−7.84	−8.91	−11.50	−6.33	51	−8.56	−10.23	−12.82	−7.64
48 months	40	−8.69	−10.08	−12.96	−7.21	38	−7.89	−9.56	−12.44	−6.67
60 months	21	−5.31	−8.43	−12.06	−4.81	17	−7.99	−7.88	−11.79	−3.98

**Table C1:** FACT-ES Total score observed score

	Chemotherapy plus Goserelin						Chemotherapy					
	N	Mean	SE	Median	Min	Max	N	Mean	SE	Median	Min	Max
Baseline	95	173.76	2.31	180.00	93.00	211.76	104	173.72	2.37	177.00	89.83	213.00
3 months	91	148.91	3.29	150.72	77.00	216.00	100	150.14	3.14	149.50	86.00	211.00
6 months	84	148.21	3.62	151.50	66.00	215.00	101	147.52	3.50	149.00	68.00	217.00
12 months	83	163.52	3.37	158.00	80.00	215.00	94	163.05	3.19	168.50	87.00	213.00
18 months	78	166.59	3.48	170.75	80.17	216.00	89	167.29	3.58	177.00	62.06	212.00
24 months	74	167.63	3.65	174.00	101.00	215.00	87	164.52	3.74	169.00	54.00	216.00
36 months	56	171.46	4.71	185.00	80.77	216.00	57	168.48	4.46	177.00	66.00	215.00
48 months	40	168.39	5.66	177.50	75.08	215.00	42	171.66	5.55	182.06	73.00	219.00
60 months	21	180.12	7.48	190.00	99.56	218.00	18	175.86	8.32	185.83	76.00	207.00

**Table C2:** FACT-ES Total score change from baseline

	Chemotherapy plus Goserelin					Chemotherapy				
		Observed	Estimated				Observed	Estimated		
	N	Mean	Mean	95% lcl	95% ucl	N	Mean	Mean	95% lcl	95% ucl
3 months	90	−24.55	−24.06	−29.65	−18.47	94	−24.27	−24.31	−29.70	−18.91
6 months	83	−25.20	−26.27	−31.95	−20.59	92	−25.77	−26.31	−31.72	−20.89
12 months	82	−8.67	−9.66	−15.37	−3.95	85	−11.15	−12.74	−18.25	−7.23
18 months	77	−7.73	−9.11	−14.92	−3.30	81	−6.62	−8.91	−14.51	−3.31
24 months	73	−6.27	−7.07	−12.97	−1.17	80	−8.62	−10.27	−15.91	−4.63
36 months	54	−4.60	−6.69	−13.02	−0.36	51	−3.44	−7.60	−13.93	−1.27
48 months	39	−5.99	−9.33	−16.37	−2.30	38	−0.38	−6.49	−13.54	0.57
60 months	20	7.72	−2.61	−11.56	6.35	17	−2.32	−3.64	−13.13	5.85

## Appendix A

ORs less than 1 imply a better QoL for the treatment including goserelin: i.e. the odds of experiencing the symptom were lower in the group treated with goserelin.
